# A autopercepção da imagem corporal dos adolescentes brasileiros nos anos de 2009 a 2019 segundo a *Pesquisa Nacional de Saúde do Escolar* (PeNSE)

**DOI:** 10.1590/0102-311XPT154723

**Published:** 2024-09-09

**Authors:** Juliana Teixeira Antunes, Jéssica Vieira Lisboa

**Affiliations:** 1 Instituto Federal do Norte de Minas Gerais, Montes Claros, Brasil.

**Keywords:** Saúde Mental, Imagem Corporal, Saúde do Adolescente, Política Pública, Adolescente, Mental Health, Body Image, Adolescent Health, Public Policy, Adolescent, Salud Mental, Imagen Corporal, Salud del Adolescente, Política Pública, Adolescente

## Abstract

Este estudo analisa a prevalência da autopercepção da imagem corporal relatada pelos adolescentes entre os anos de 2009 e 2019 segundo sexo e região com base na *Pesquisa Nacional de Saúde do Escolar* (PeNSE). Foi realizada uma análise epidemiológica, descritiva de série temporal com medidas de prevalência e tendência de como os adolescentes se percebem em relação ao próprio corpo, conforme os dados fornecidos pelas edições da PeNSE nos anos de 2009 a 2019. A prevalência dos adolescentes que se consideravam “normais” atingiu 47,6% (IC95%: 46,1-49,1) em 2019, representando uma diferença negativa de 12,5 pontos percentuauis (p.p.) e uma variação de 20,7% em relação ao ano de 2009. Em 2019, 31,4% (IC95%: 30,0-32,9) dos meninos relataram sentir-se magros ou muito magros, representando uma diferença de 8,4p.p. em relação a 2009. Já as meninas tiveram uma prevalência de 28,6% (IC95%: 26,1-31,1) em sentir-se gordas ou muito gordas no ano de 2019, representando uma variação de 7,3p.p. em relação a 2009. Nos últimos anos, houve uma mudança na autopercepção corporal dos adolescentes, com redução nas prevalências daqueles que se consideravam “normais” e um aumento entre aqueles que se consideravam magros ou muito magros para o sexo masculino e gordos ou muito gordos para o sexo feminino. Tais resultados apontam para a importância de investigar as consequências da autopercepção magra ou muito magra e gorda ou muito gorda na vida dos adolescentes.

## Abstract (en)

This study analyzes the self-perception of body image reported by adolescents from 2009 to 2019 according to sex and region, based on the *Brazilian National Survey of School Health* (PeNSE). An epidemiological, descriptive time-series analysis was carried out with prevalence and trend measures of how adolescents perceive themselves in relation to their bodies according to the data provided by the PeNSE surveys from 2009 to 2019. The prevalence of adolescents who considered themselves normal reached 47.6% (95%CI: 46.1-49.1) in 2019, representing a negative difference of 12.5 percentage points (p.p.) and a variation of 20.7% compared to 2009. In 2019, 31.4% (95%CI: 30.0-32.9) of boys reported feeling thin or very thin, a difference of 8.4p.p. compared to 2009. On the other hand, 28.6% (95%CI: 26.1-31.1) of girls perceived themselves as fat or very fat in 2019, representing a variation of 7.3p.p. compared to 2009. In recent years, there has been a change in the body self-perception of adolescents, with a reduction in the prevalence of those who consider themselves normal and an increase among those who consider themselves thin or very thin for males and fat or very fat for females. These results indicates the importance of investigating the consequences of perceiving oneself as thin or very thin and fat or very fat in the lives of adolescents.

## Abstract (es)

Este estudio analiza la prevalencia de la autopercepción de la imagen corporal reportada por adolescentes entre el 2009 y el 2019 según el sexo y la región con base en la *Encuesta Nacional de Salud del Escolar* (PeNSE). Se realizó un análisis epidemiológico, descriptivo de serie temporal con medidas de prevalencia y tendencia de la manera en que los adolescentes se perciben con relación a su cuerpo según los datos proporcionados por las ediciones de la PeNSE del 2009 al 2019. La prevalencia de los adolescentes que se consideraban normales alcanzó el 47,6% (IC95%: 46,1-49,1) en el 2019, lo que representa una diferencia negativa de 12,5 puntos porcentuales (p.p.) y una variación del 20,7% con relación al año del 2009. En el 2019, el 31,4% (IC95%: 30,0-32,9) de los chicos refirieron sentirse delgados o muy delgados, lo que representa una diferencia de 8,4p.p. respecto al 2009. A su vez, las chicas tuvieron una prevalencia del 28,6% (IC95%: 26,1-31,1) en sentirse gordas o muy gordas en el 2019, lo que representa una variación de 7,3p.p. respecto al 2009. En los últimos años, se produjo un cambio en la autopercepción corporal de los adolescentes con una reducción en las prevalencias de los que se consideraban normales y un aumento entre los que se consideraban delgados o muy delgados para el sexo masculino y gordas o muy gordas para el sexo femenino. Estos resultados apuntan a la importancia de investigar las consecuencias de la autopercepción de ser delgado o muy delgado y gordo o muy gordo en la vida de los adolescentes.

## Introdução

A adolescência é uma fase marcada por mudanças físicas, psicológicas e sociais desencadeadas pelas modificações neuroquímicas e neurotransmissoras do sistema nervoso. Durante essa fase, o córtex cerebral, região responsável pelo pensamento e raciocínio do ser humano, ainda em desenvolvimento, predispõe os jovens a situações de vulnerabilidade em sua saúde ^1^. Assim, as inseguranças diante das transformações sofridas nesse período tornam-se prevalentes, desencadeando, muitas vezes, problemas de saúde mental como ansiedade, medo, nervosismo, estresse, exposição à pobreza e à violência ^1^ ^,^ ^2^.

Para a construção de uma personalidade madura e segura, o adolescente necessita de uma boa relação consigo mesmo, de uma boa autoimagem e autoestima ^3^. Em decorrência da valorização da beleza corporal, das mudanças físicas e psicológicas provocadas pela adolescência e do desejo de pertencer a um grupo, os jovens estão mais vulneráveis a assumirem comportamentos de risco à saúde, como dietas e atividades físicas sem acompanhamento profissional ^4^ ^,^ ^5^, preocupações excessivas com o peso e consumo de álcool. Tais comportamentos revelam a percepção que o adolescente tem do seu corpo e podem influenciar negativamente em seu desenvolvimento saudável ^6^ ^,^ ^7^.

Assim, a autopercepção negativa do corpo pode desencadear diversos problemas na saúde do indivíduo, incluindo o desenvolvimento de transtornos mentais ^6^ ^,^ ^7^. Autores indicam que jovens com uma percepção negativa do corpo relataram ter mais dificuldades para dormir à noite, maior nervosismo, estresse, depressão, baixa autoestima e pior qualidade de vida ^4^. Portanto, investigar a autopercepção da imagem corporal entre os adolescentes torna-se uma estratégia fundamental para garantir a saúde dessa faixa etária, fomentando ações de promoção da saúde física e mental em diferentes ambientes e culturas ^6^.

A adolescência é uma fase que requer atenção e investimento em políticas públicas de promoção da saúde, além da vigilância de comportamentos e fatores de risco e proteção à saúde ^8^. Além disso, autores apontam a necessidade de estudos nacionais que investiguem a autopercepção da imagem corporal entre os adolescentes ^5^, identificando sua distribuição entre os adolescentes brasileiros e promovendo mais estudos sobre sua relação com a saúde dessa população.

Portanto, este artigo apresenta de forma inédita uma análise temporal de abrangência nacional da autopercepção da imagem corporal da população de adolescentes brasileiros nos últimos anos, segundo o sexo e a região. O objetivo é identificar as diferenças nas prevalências da autopercepção da imagem corporal relatada pelos adolescentes entre os anos de 2009 e 2019, conforme a *Pesquisa Nacional de Saúde do Escolar* (PeNSE).

## Metodologia

Trata-se de um estudo epidemiológico de série temporal, com base nas prevalências e intervalos de confiança disponibilizados pela PeNSE no *site* do Instituto Brasileiro de Geografia e Estatística (IBGE) nos anos de 2009, 2012, 2015 e 2019. A PeNSE é uma pesquisa de abrangência nacional feita pelo IBGE, em parceria com o Ministério da Saúde e com o apoio do Ministério da Educação, que fornece informações para o sistema de vigilância de fatores de risco e proteção para a saúde dos escolares, com dados atualizados sobre a prevalência e a distribuição desses fatores nessa população ^9^.

Na sua primeira edição, elaborada em 2009, foi feita uma amostragem de conglomerados em dois estágios, em que as escolas brasileiras corresponderam às unidades primárias de amostragem e as turmas do 9º ano do Ensino Fundamental diurno das escolas selecionadas corresponderam às unidades secundárias de amostragem. Assim, a amostra de adolescentes foi composta por todos os escolares das turmas unidades secundárias de amostragem A selecionadas na amostra de escolas unidades primárias de amostragem. As escolas com turmas do 9º ano do Ensino Fundamental, privadas ou públicas (federais, estaduais ou municipais), foram estratificadas conforme a localização geográfica, correspondendo às capitais das Unidades da Federação (UF), totalizando 27 estratos ^9^. Portanto, a amostra da edição da PeNSE 2009 contou com a participação de escolares brasileiros do 9º ano do Ensino Fundamental frequentando a escola nas capitais e no Distrito Federal.

Já em 2012, em sua segunda edição, a amostra da PeNSE abrangeu diversos domínios geográficos, apresentando, além dos 26 municípios das capitais e do Distrito Federal, cada uma das cinco grandes regiões brasileiras e o Brasil em sua totalidade ^9^. Em 2015, a PeNSE analisou dois planos amostrais distintos, sendo um representativo de escolares frequentando o 9º ano do Ensino Fundamental diurno (Amostra 1) e outro de escolares de 13 a 17 anos de idade (Amostra 2) ^9^. As UF foram incorporadas à Amostra 1, ampliando sua abrangência geográfica, e a Amostra 2 ficou restrita ao Brasil e às grandes regiões ^8^. No ano de 2019, a PeNSE trabalhou com uma amostra única de adolescentes de 13 a 17 anos, equivalente à Amostra 2 da edição de 2015 ^9^. O plano amostral de 2019 também foi por conglomerados em dois estágios, sendo o primeiro correspondente às turmas e o segundo às escolas, abrangendo os níveis geográficos: Brasil, Grandes Regiões, UF, Municípios das Capitais ^9^. Mais informações sobre o processo de amostragem das edições da PeNSE podem ser consultadas no site, de acesso público (https://metadados.ibge.gov.br/consulta/estatisticos/operacoes-estatisticas/AA).

A coleta de dados da PeNSE acontece por questionários aplicados por aparelhos eletrônicos como o assistente digital pessoal (PDA) e *smartphone* aos estudantes. Para este estudo, foram analisadas as prevalências das variáveis de interesse obtidas de amostras probabilísticas de estudantes do 9º ano do Ensino Fundamental matriculados e com frequência regular em escolas públicas e privadas de todo o território nacional nos anos investigados. A variável dependente compreende os adolescentes que se consideram magros ou muito magros; normais; gordos ou muito gordos, segundo as respostas à pergunta: “Quanto ao seu corpo, você se considera: muito magro(a), magro(a), normal, gordo(a), muito gordo(a)”, realizada nas edições de 2009, 2012, 2015 e 2019 da PeNSE. As variáveis independentes deste estudo abrangeram os anos da pesquisa (2009, 2012, 2015 e 2019), o sexo (masculino, feminino) e as macrorregiões brasileiras (Norte, Nordeste, Sudeste, Sul e Centro-oeste). Na análise segundo as macrorregiões, o ano de 2009 não foi analisado devido à ausência de informações sobre os indicadores nesse período, pois a amostra da PeNSE 2009 foi estratificada, conforme sua localização geográfica, referente às capitais das UF e ao Distrito Federal. Já para 2019, devido à complexidade e ao tamanho do banco de dados, tornou-se impossível sua análise no programa Excel (https://products.office.com/), programa disponível para análise deste estudo.

Os dados foram analisados pelo pacote estatístico Excel, versão 16.7. Primeiramente, as prevalências dos desfechos e respectivos intervalos de 95% de confiança (IC95%) foram estimados segundo as variáveis independentes. Em seguida, foi feita a regressão polinomial para a análise de tendência e quantificou-se a variação anual nas prevalências entre 2009 e 2019. 

As edições da PeNSE foram aprovadas pela Comissão Nacional de Ética em Pesquisa segundo os pareceres nº 11.537 em 2009; nº 16.805 em 2012; nº 1.006.467 em 2015 e nº 3.249.268 em 2019.

## Resultados

**Figura 1:**
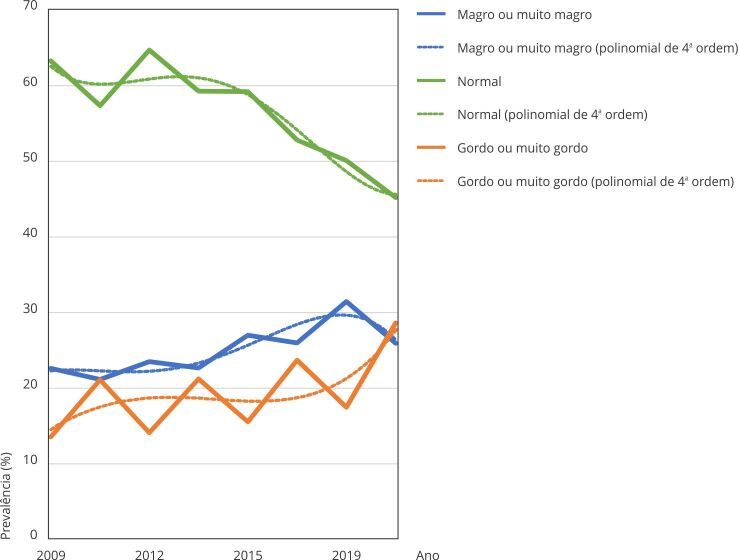
Tendência da autopercepção da imagem corporal dos adolescentes brasileiros. *Pesquisa Nacional de Saúde do Escolar* (PeNSE), Brasil, 2009-2019.

O número de adolescentes escolares do 9º ano do Ensino Fundamental avaliados em cada ano da pesquisa foi de 63.411 em 2009; 109.104 em 2012; 102.301 em 2015 e 574.799 em 2019 ^9^ ^,^ ^10^. Entre os anos de 2009 e 2019, houve uma mudança na autopercepção da imagem corporal entre os adolescentes brasileiros, observando um aumento de 22,1% (IC95%: 21,5-22,7) em 2009 para 28,6% (IC95%: 27,6-29,7) em 2019 nas prevalências de adolescentes que se consideram magros ou muito magros, correspondendo a uma diferença de 6,5 pontos percentuais (p.p.) (Tabela 1). A prevalência daqueles adolescentes que se consideravam gordos ou muito gordos foi de 17,7% (IC95%: 17,2-18,3) em 2009 e 23,2% (IC95%: 22,2-24,2) em 2019, com uma diferença de 5,5p.p. entre os anos investigados. Para os adolescentes que se consideravam “normais”, a prevalência foi de 60,1% (IC95%: 59,5-60,8) em 2009 e 47,6% (IC95%: 46,1-49,1) em 2019, com uma diferença de -12,5p.p. Observa-se uma maior variação anual na prevalência entre aqueles adolescentes que se consideravam gordos ou muito gordos, atingindo 31% de aumento entre os anos 2009 e 2019. A Figura 1 nos mostra uma tendência decrescente em perceber-se “normal” (R^2^ = 0,9012) e magro ou muito magro (R^2^ = 0,8067) e uma tendência crescente entre aqueles adolescentes que se percebiam gordos ou muito gordos (R^2^ = 0,5317).

**Tabela 1:** Prevalência, diferença em pontos percentuais (p.p.) e variação percentual da autopercepção da imagem corporal de adolescentes brasileiros para o Brasil e segundo o sexo. *Pesquisa Nacional de Saúde do Escolar* (PeNSE), Brasil, 2009-2019.

	2009 % (IC95%)	2012 % (IC95%)	2015 % (IC95%)	2019 % (IC95%)	Diferença (p.p.)	Variação (%)
Magro ou muito magro						
Total	22,1 (21,5-22,7)	21,9 (21,0-22,7)	25,8 (25,3-26,3)	28,6 (27,6-29,7)	6,5	29,4
Masculino	23,0 (22,1-23,8)	22,2 (21,3-23,0)	26,2 (25,5-26,9)	31,4 (30,0-32,9)	8,4	36,5
Feminino	21,4 (20,6-22,1)	21,6 (20,6-22,5)	25,4 (24,6-26,1)	25,9 (24,4-27,5)	4,5	21,0
Normal						
Total	60,1 (59,5-60,8)	61,9 (61,6-62,1)	55,9 (55,3-56,5)	47,6 (46,1-49,1)	-12,5	-20,7
Masculino	63,3 (62,3-64,3)	64,7 (64,3-65,1)	59,2 (58,4-60,0)	50,1 (48,0-52,2)	-13,2	-20,8
Feminino	57,3 (56,4-58,3)	59,3 (58,8-59,7)	52,8 (52,0-53,6)	45,2 (41,9-48,5)	-12,1	-21,1
Gordo ou muito gordo						
Total	17,7 (17,2-18,3)	16,3 (15,4-17,1)	18,3 (17,9-18,8)	23,2 (22,2-24,2)	5,5	31,0
Masculino	13,8 (13,1-14,5)	13,1 (12,0-14,2)	14,6 (14,0-15,2)	17,5 (15,8-19,2)	3,7	26,8
Feminino	21,3 (20,5-22,1)	19,1 (18,2-20,0)	21,8 (21,2-22,5)	28,6 (26,1-31,1)	7,3	34,2

IC95%: intervalo de 95% de confiança.

Fonte: elaborado própria.

A comparação dos indicadores de autopercepção da imagem corporal entre os sexos apresentou diferenças entre o feminino e masculino, mostrando maiores prevalências para os meninos em se perceberem “normais” entre os anos de 2009 e 2019 (Tabela 1). A diferença entre os anos investigados de perceber-se “normal” para o sexo masculino foi de -13,2p.p., representando uma variação de -20,8% em relação ao ano de 2009. Os meninos também apresentaram maiores prevalências em perceberem-se magros ou muito magros, com uma diferença de 8,4p.p. e uma variação de 36,5% de aumento em relação ao ano de 2009, valores que correspondem quase ao dobro dos apresentados pelo sexo feminino. Já para o indicador de se perceberem gordos ou muito gordos, o sexo masculino apresentou prevalências sempre inferiores às do sexo feminino, com uma diferença de 3,7p.p. entre os anos investigados, sendo essa também inferior à apresentada pelo sexo feminino (7,3p.p.) (Tabela 1).

Para o sexo feminino, observa-se uma redução nas prevalências do indicador de perceber-se “normal” em relação ao seu corpo entre os anos de 2009 e 2019, com uma variação de -12,1p.p. (o que equivale a -21,1%) (Tabela 1). Em relação aos meninos, as meninas apresentaram menor prevalência nos indicadores de perceber-se magras ou muito magras, com uma diferença de 4,5pp desse indicador, correspondendo quase à metade da diferença apresentada pelo sexo masculino (Tabela 1). As adolescentes do sexo feminino também apresentaram maiores prevalências em se perceberem gordas ou muito gordas, atingindo uma prevalência de 28,6% no ano de 2019 e uma diferença de 7,3p.p. (o que equivale a 34,2%) entre os anos de 2009 e 2019 (Tabela 1).

**Figura 2:**
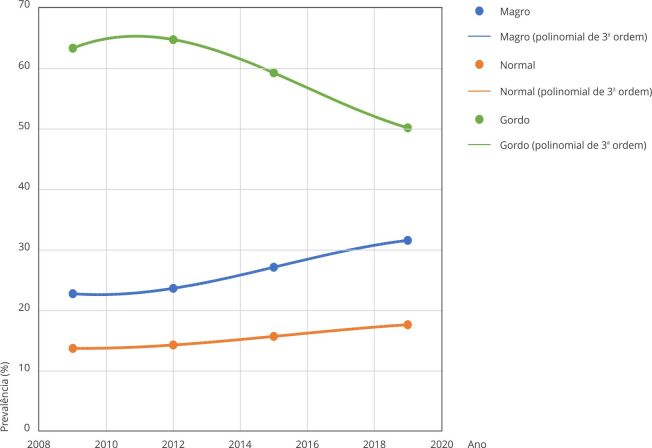
Tendência da autopercepção da imagem corporal dos adolescentes do sexo masculino. *Pesquisa Nacional de Saúde do Escolar* (PeNSE), Brasil, 2009-2019.

**Figura 3:**
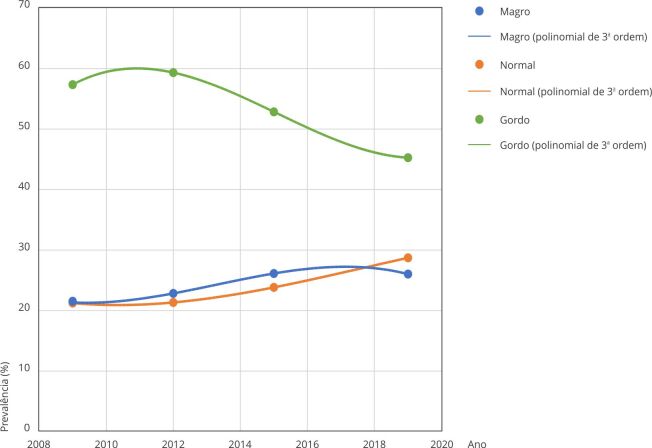
Tendência da autopercepção da imagem corporal dos adolescentes do sexo feminino. *Pesquisa Nacional de Saúde do Escolar* (PeNSE), Brasil, 2009-2019.

As linhas de tendência apresentadas na Figura 2 mostram uma queda na autopercepção da imagem corporal dos adolescentes do sexo masculino em perceberem-se “normais” (R^2^ = 1) e um aumento nos indicadores de perceberem-se gordos ou muito gordos (R^2^ = 1) e magros ou muito magros (R^2^ = 1) no período estudado. Para o sexo feminino, as linhas de tendência observadas na Figura 3 mostram um leve aumento na autopercepção da imagem corporal entre as meninas que se percebiam gordas ou muito gordas 152 (R^2^ = 1) e “normais” (R^2^ = 1) em relação ao seu corpo, e uma tendência decrescente na prevalência em perceberem-se magras (R^2^ = 1). 

A Tabela 2 nos mostra uma pequena diferença entre as prevalências da autopercepção da imagem corporal dos adolescentes nas regiões brasileiras em 2012 e 2015. Porém, é possível notar que para o ano de 2012, a Região Sul apresentou menor prevalência (19,6%, IC95%: 18,5-20,7) entre os adolescentes que se percebiam magros ou muito magros e a maior prevalência (19,6%, IC95%: 18,9-20,2) de adolescentes que se percebiam gordos ou muito gordos. Já as regiões Norte e Nordeste apresentaram as maiores prevalências de adolescentes que se percebiam “normais” quanto ao próprio corpo. Para o ano de 2015, a Região Nordeste alcançou a maior prevalência de adolescentes que se percebiam magros ou muito magros (26,3%, IC95%: 25,7-26,9); a Região Sul, a maior prevalência daqueles que se percebiam gordos ou muito gordos (22,3%, IC95%: 21,1-23,6); e a Região Norte, a maior prevalência de adolescentes que se percebiam “normais” quanto ao próprio corpo (59,3%, IC95%: 58,2-60,3). A comparação da variação da prevalência dos indicadores de autopercepção da imagem corporal entre as regiões no período investigado mostra que a Região Sul apresentou maior variação relativa na prevalência dos adolescentes que se percebiam magros ou muito magros (25%) e “normais” (-12,6%). A Região Centro-oeste apresentou maior variação relativa na prevalência dos adolescentes em perceberem-se gordos ou muito gordos (17,6%) em relação ao ano de 2012. A Região Norte apresentou a menor variação relativa (-7,4%) no indicador de perceber-se “normal” em relação ao próprio corpo no período investigado. 

**Tabela 2:** Prevalência, diferença em pontos percentuais (p.p.) e variação percentual da autopercepção da imagem corporal de adolescentes brasileiros segundo a macrorregião no período de 2012-2015. *Pesquisa Nacional de Saúde do Escolar* (PeNSE), Brasil.

	2012 % (IC95%)	2015 % (IC95%)	Diferença (p.p.)	Variação (%)
Magro ou muito magro				
Norte	22,4 (21,6-23,2)	25,6 (24,6-26,5)	3,2	14,2
Nordeste	22,3 (21,5-23,2)	26,3 (25,7-26,9)	4	17,9
Sudeste	22,4 (20,6-24,1)	26,0 (25,0-27,0)	3,6	16,0
Sul	19,6 (18,5-20,7)	24,5 (23,4-25,7)	4,9	25,0
Centro-oeste	21,3 (20,3-22,3)	24,5 (23,7-25,3)	3,2	15,0
Normal				
Norte	64,1 (63,5-64,7)	59,3 (58,2-60,3)	-4,8	-7,4
Nordeste	64,5 (64,3-64,7)	59,1 (58,3-59,9)	-5,4	-8,3
Sudeste	60,2 (59,8-60,7)	53,9 (52,7-55,0)	-6,3	-10,4
Sul	60,8 (60,3-61,3)	53,1 (51,6-54,6)	-7,7	-12,6
Centro-oeste	62,2 (61,4-63,0)	56,2 (55,1-57,2)	-6,0	-9,6
Gordo ou muito gordo				
Norte	13,5 (13,1-13,9)	15,2 (14,4-15,9)	1,7	12,5
Nordeste	13,1 (12,4-13,9)	14,6 (14,0-15,2)	1,5	11,4
Sudeste	17,4 (15,3-19,5)	20,1 (19,3-21,0)	2,7	15,5
Sul	19,6 (18,9-20,2)	22,3 (21,1-23,6)	2,7	13,7
Centro-oeste	16,4 (14,8-18,1)	19,3 (18,5-20,2)	2,9	17,6

IC95%: intervalo de 95% de confiança.

Fonte: elaborado própria.

## Discussão

A aparência física é algo extremamente valorizado nas diversas culturas ^4^. Para os adolescentes, a autoimagem pode representar sua personalidade ^3^ e seus hábitos de vida, incluindo aqueles prejudiciais à saúde, como consumo excessivo de álcool, sedentarismo ^6^ ^,^ ^7^, hábitos alimentares errôneos e práticas de atividade física inadequadas ^11^ ^,^ ^12^, revelando os cuidados à saúde e ao bem-estar psicológico dos adolescentes ^11^ ^,^ ^13^. Nos últimos anos, observa-se uma redução na prevalência de adolescentes que mantêm uma autopercepção considerada normal de sua imagem corporal. Essa tendência levanta preocupações quanto à saúde dessa população, uma vez que a imagem corporal está intrinsecamente ligada a práticas e comportamentos relacionados ao corpo ^14^. Isso inclui a adoção de medidas de risco para a saúde, como o uso de fórmulas e laxantes para alterações no peso ou ganho de massa muscular, padrões alimentares inadequados e a prática de atividades físicas extenuantes ^14^. Além disso, a redução na prevalência de adolescentes que se consideravam dentro da normalidade em relação ao próprio corpo pode refletir uma correspondência com seu estado nutricional ou índice de massa corporal (IMC), indicando um aumento nas medidas antropométricas dos adolescentes no período analisado ^14^. Estudos apresentaram uma maior proporção de alunos com baixo peso que se percebiam como “magros”, enquanto aqueles com peso adequado se viam como “normais”, e os que apresentavam excesso de peso associavam-se à percepção de si mesmos como “gordos” ^8^.

A autopercepção da imagem corporal de um adolescente, quando em desacordo com seu estado nutricional real, pode revelar uma preocupação exacerbada com o peso e modificações no seu IMC, podendo resultar na adoção de comportamentos prejudiciais à saúde dos adolescentes ^5^ ^,^ ^6^ ^,^ ^7^. Uma autopercepção da imagem corporal divergente do estado nutricional real dos adolescentes relaciona-se à influência que os padrões culturais de cada época exercem na percepção do corpo ideal dos adolescentes ^4^ ^,^ ^5^ ^,^ ^6^ ^,^ ^7^ ^,^ ^8^ ^,^ ^9^ ^,^ ^10^ ^,^ ^11^. Assim, a autopercepção da imagem corporal entre adolescentes sofre influência de diversos fatores, como as redes sociais, as propagandas de televisão, as revistas, os jogos de videogame da atualidade ^4^, fatores sociodemográficos como escolaridade materna, situação administrativa da escola, idade e etnia ^11^, que contribuem para uma percepção de magreza, “normalidade” ou obesidade divergente entre os indivíduos.

Meninos e meninas demonstram diferenças na autopercepção da imagem corporal que podem ser influenciadas por diversas condições. Estudo indica, por exemplo, uma maior prevalência na distorção da imagem corporal entre as meninas com peso adequado que se viam gordas e uma maior prevalência entre os meninos que apresentavam estado nutricional adequado em atitudes de tentar ganhar peso ^8^. Corroborando os resultados apresentados nesse estudo, autores apontam um desejo das meninas de serem mais magras, enquanto os meninos desejam ser mais fortes e musculosos ^6^ ^,^ ^7^ ^,^ ^8^ ^,^ ^9^ ^,^ ^10^ ^,^ ^11^ ^,^ ^12^ ^,^ ^13^. Concomitantemente, há um incentivo à prática de atividade física e esportes para o ganho de massa muscular entre os meninos e um maior estímulo a práticas de controle ou perda de peso entre as meninas, o que pode contribuir com a maior prevalência do sexo feminino em perceber-se gorda ou muito gorda ^13^. Portanto, a maior prevalência em perceber-se gorda verificada entre as meninas pode resultar em um risco para a saúde das adolescentes, pois um estudo revelou que entre as meninas que se consideravam com estado nutricional adequado ou com excesso de peso, notou-se uma maior prevalência de tentativas de perder peso ^8^, como a adoção de hábitos alimentares prejudiciais à saúde. O sobrepeso e a obesidade, juntamente com outros fatores como: o aumento da idade; o maior estadiamento de maturação sexual e os altos níveis socioeconômicos entre as meninas aumentam as chances de uma percepção negativa do seu corpo e o desejo de diminuir a silhueta corporal ^15^. Um estudo desenvolvido entre adultos também apontou uma forte relação entre a insatisfação com a imagem corporal e o sexo feminino ^7^, demonstrando a importância da infância e da adolescência como períodos em que se adquirem hábitos que repercutirão nas fases de vida adulta ^11^.

Assim, a autopercepção da imagem corporal como “normal” pode revelar o cuidado que cada indivíduo tem com o próprio peso. Um estudo mostrou que há uma maior proporção de autoimagem “normal” entre os meninos com excesso de peso e que esses não faziam nenhuma atividade para perda de peso ^8^.

A região geográfica também pode interferir na prevalência da autopercepção da imagem corporal entre os adolescentes. Tomando a relação entre a autopercepção da imagem corporal e a real classificação do estado nutricional dos adolescentes ^14^, as diferenças apresentadas na variação anual dos indicadores por região podem revelar uma influência dos fatores nutricionais, socioeconômicos, ambientais e genéticos no estado nutricional e, consequentemente, na autopercepção da imagem corporal dos adolescentes brasileiros. As regiões com melhores condições de vida como Sul e Sudeste garantem maior ganho de estatura e peso entre os adolescentes em comparação com as regiões Norte e Nordeste ^16^ ^,^ ^17^, o que pode justificar a menor prevalência entre os adolescentes sulistas em se perceberem magros ou muito magros e sua maior prevalência em perceberem-se gordos ou muito gordos. Há também uma autopercepção de considerar-se mais altas e uma maior influência de ideais de beleza e saúde mais magros entre as adolescentes da Região Sul ^17^, o que pode influenciar a autopercepção da imagem corporal das adolescentes que acabam se considerando gordas ou muito gordas, assumindo comportamentos para alcançarem o peso ideal. Na Região Norte, meninos e meninas apresentam a menor média de estatura e a menor prevalência para excesso de peso e para comportamentos sedentários ^17^ ^,^ ^18^, o que pode contribuir para a maior prevalência desses adolescentes em se perceberem “normais” e para a menor variação desse indicador entre as regiões brasileiras nos anos de 2012 e 2015. Em contraponto, a Região Centro-oeste apresentou maior variação e aumento na prevalência de adolescentes que se percebiam gordos ou muito gordos, sendo uma região caracterizada por indivíduos com maiores parâmetros de medidas antropométricas do Brasil ^19^. Além desses, fatores socioeconômicos, como: renda familiar, pertencer ao tercil socioeconômico mais baixo e menor escolaridade materna; estão associados à menor estatura, ao excesso de peso e ao sedentarismo dos adolescentes brasileiros, refletindo no estado nutricional dos adolescentes, no seu crescimento saudável e na autopercepção de sua imagem corporal ^17^ ^,^ ^18^. Com o passar do tempo, nota-se um aumento da prevalência dos adolescentes que se percebiam gordos em relação à própria imagem corporal. Diversos fatores podem contribuir para essa autopercepção, como o real aumento do IMC ^12^, a valorização de corpos magros, musculosos e definidos e a influência que os agravos à saúde física e psicológica exercem na imagem corporal dos indivíduos ^10^.

A autopercepção da imagem corporal é um importante indicador da saúde do adolescente, contribuindo para a identificação dos fatores de risco ao desenvolvimento e de hábitos prejudiciais à saúde dessa população ^5^. Há uma necessidade de criar políticas públicas que permitam discussões e reflexões sobre a importância da percepção corporal na saúde dos adolescentes, viabilizando ações e programas nas escolas e famílias ^13^.

Este estudo apresenta algumas limitações, como a ausência de dados segundo a macrorregião nos anos de 2009 e 2019, comprometendo a interpretação da série histórica para esse indicador, assim como a impossibilidade de determinar os fatores causais da diferença na prevalência da autopercepção da imagem corporal entre os sexos, região e anos investigados. Também há falta de representação na amostra de adolescentes que não estão matriculados e frequentando as escolas públicas ou privadas do território brasileiro, que, apesar de representarem uma pequena porcentagem da população, poderiam ampliar os resultados mostrados neste artigo para outras realidades. As classificações corporais utilizadas neste estudo são limitadas à interpretação individual, não levando em consideração fatores importantes como os aspectos comportamentais da imagem corporal ^14^, o estado nutricional, o IMC, a maturação sexual do adolescente, que apresentam grande influência nas dimensões e proporções corporais dos adolescentes ^8^ e na autopercepção da própria imagem corporal. Assim, torna-se importante elaborar novos estudos que investiguem os fatores associados à autopercepção da imagem corporal dos adolescentes e sua relação com a saúde física e mental dessa população.

## Conclusão

Os resultados deste estudo indicam uma redução na prevalência de adolescentes que se percebem como “normais”, enquanto se observa uma tendência de aumento da prevalência daqueles que se viam como magros ou muito magros e como gordos ou muito gordos. Adicionalmente, destaca-se uma diferença na autopercepção da imagem corporal entre os sexos, evidenciando, para o sexo masculino, maior prevalência de percepção de estar magro ou muito magro e maior aumento (relativo e em p.p.) nessa prevalência em questão; e, para o sexo feminino, maior prevalência de percepção de estar gorda ou muito gorda e maior aumento (relativo e em p.p.) dessa prevalência em questão.

Durante o período de 2012 a 2015, as regiões com melhores condições de vida apresentaram maior prevalência de adolescentes que se percebiam magros ou gordos, enquanto os adolescentes da Região Norte demonstraram uma menor variação na percepção de estar “normal”. Esses resultados sinalizam a necessidade de investigar as repercussões da autopercepção de magreza ou de gordura na vida dos adolescentes, incluindo comportamentos, padrões estéticos e dimensões corporais reais.

Observa-se, ainda, que a variação da prevalência da autopercepção da imagem corporal entre os adolescentes pode ser influenciada pelo sexo e pela região, ressaltando a importância da implementação de estratégias diferenciadas para promover o reconhecimento de um corpo considerado “normal” e saudável entre os adolescentes brasileiros.
